# A Split-Gate 4H-SiC MOSFET with a Raised Central Implant and a Stepped Schottky Contact

**DOI:** 10.3390/mi17101151

**Published:** 2026-09-30

**Authors:** Kai Xiao, Chaojun Ming, Shuming Tan, Yansheng Zou, Qian Chen, Han Xu, Kai Zheng

**Affiliations:** 1 CSG EHV Electric Power, Research Institute, China Southern Power Grid Company Limited, Guangzhou 510663, China; xiaokaiehv@163.com (K.X.); tanshm@foxmail.com (S.T.); zoulucky@foxmail.com (Y.Z.); paschen@163.com (Q.C.); 2 College of Optoelectronic Engineering and Key Laboratory of Optoelectronic Technology & Systems Education Ministry of China, Chongqing University, Chongqing 400044, China; mingchaojun@163.com; 3 China Southern Power Grid EHV Power Transmission Company, Guangzhou 510663, China; m18813296519_2@163.com; 4 State Key Laboratory of Power Transmission Equipment Technology, Chongqing University, Chongqing 400044, China; 5 Space Science and Technology Research Institute, Chongqing University, Chongqing 400044, China

**Keywords:** 4H-SiC MOSFET, split-gate, central implanted region, Schottky contact, gate oxide electric field, reverse recovery

## Abstract

To address the tradeoff between the gate oxide electric field and specific on-resistance in split-gate 4H-SiC MOSFETs, as well as the poor reverse recovery performance of the parasitic body diode, a split-gate MOSFET with a raised central implanted region and a stepped Schottky contact is proposed. The raised central P-type implanted region reduces its restriction on the JFET current path; the integrated Schottky contact improves the third-quadrant performance, while the stepped geometry reduces the electric field near the Schottky contact region. TCAD simulation results show that, with a raised height of 0.33 μm, the proposed device achieves a specific on-resistance of 4.69 mΩ·cm^2^ and a maximum gate oxide electric field of 2.93 MV/cm. The high-frequency figure of merit (HFOM) is reduced to 290.46 mΩ·nC, while the reverse recovery charge is reduced to 676.00 nC/cm^2^. Compared with a conventional Schottky contact, the stepped contact reduces the electric field at the critical contact location by 62.4%. These results demonstrate that the proposed device improves the conduction, gate-charge, and reverse recovery characteristics while maintaining its breakdown capability.

## 1. Introduction

4H-SiC power MOSFETs have been widely used in high-voltage and high-frequency power conversion systems owing to their excellent conduction, switching, and blocking characteristics [1,2]. To reduce switching loss, the split-gate MOSFET has been proposed. By reducing the coupling area between the gate and drift region, the split-gate structure lowers the gate-to-drain charge and improves the HFOM [3]. Buffered gates, deep Pwells, source field plates, and P^+^ buffer layers have also been investigated to improve high-frequency performance and reduce the gate oxide electric field [4,5,6,7,8]. However, electric field crowding tends to occur at the split-gate edge in the off state [3,5,7,8]. A source-connected central P-type implanted region in the JFET region can shield the gate oxide from the electric field [6,9,10], but it also restricts carrier spreading and increases the specific on-resistance. Therefore, the split-gate MOSFET with a central implanted region still faces a tradeoff between the gate oxide electric field and on-resistance.

The parasitic PiN body diode of a SiC MOSFET has a high turn-on voltage and a large reverse recovery charge, which increase third-quadrant losses [11,12,13,14]. Monolithic integration of a Schottky barrier diode provides a majority carrier path for reverse current, suppressing body diode conduction and improving reverse recovery performance [15,16]. Previous studies have improved reverse conduction by placing Schottky contacts between split gates, along trench sidewalls, or near P-type shielding regions, as well as by introducing other low-loss integrated diodes [16,17,18,19,20,21,22,23,24,25,26]. However, high electric fields may develop at the Schottky contact under reverse blocking conditions, leading to stronger barrier lowering and increased reverse leakage. The contact structure also affects the temperature characteristics and short-circuit reliability of the device [18,19,21,22,27,28,29]. In addition, the shielding region and Schottky contact dimensions must be designed to balance JFET conduction and reverse conduction capability [21,24,30]. Meanwhile, the switching characteristics of SiC MOSFETs are also affected by gate-drive conditions, particularly the applied gate voltage [31].

To address these issues, a split-gate SiC MOSFET with a raised central implanted region and a stepped Schottky contact (RSC-MOS) is proposed. Raising the central P-type implanted region reduces its restriction on the JFET current path while retaining its shielding effect on the gate oxide. The integrated Schottky contact provides a low-voltage unipolar path for third-quadrant conduction, while the stepped geometry is introduced to reduce the electric field at the metal/SiC interface. In this paper, TCAD simulation is employed to analyze the static characteristics, gate oxide electric field, gate charge, third-quadrant characteristics, and reverse recovery characteristics of the RSC-MOS.

## 2. Device Structure and Design Optimization

**Figure 1:**
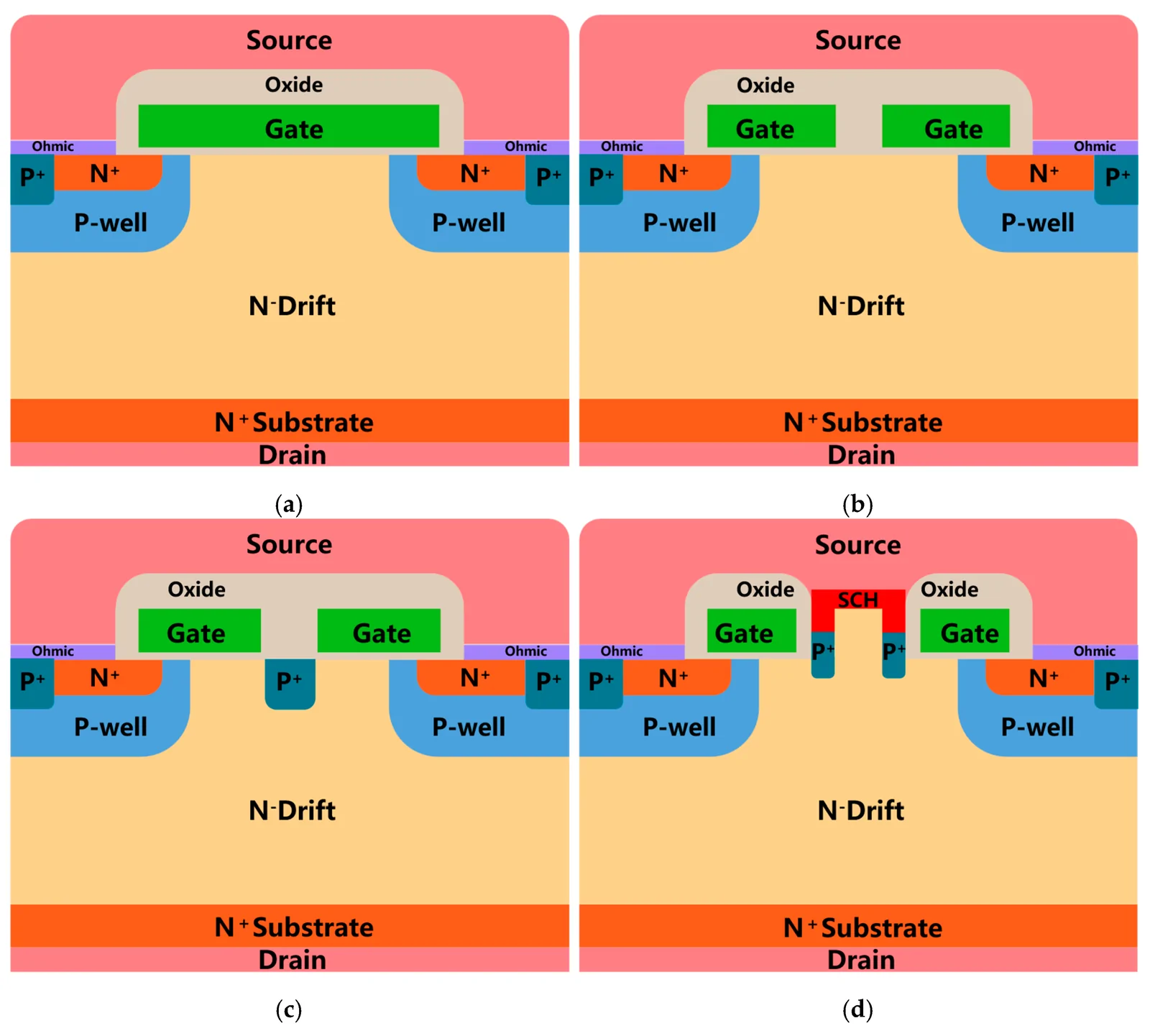
The cell cross section of (**a**) C-MOS, (**b**) SG-MOS, (**c**) SC-MOS and (**d**) RSC-MOS.

Figure 1 shows the unit cell structures of the four MOSFETs investigated in this work: the conventional planar gate MOSFET (C-MOS), split-gate MOSFET (SG-MOS), split-gate MOSFET with a central P-type implanted region (SC-MOS), and the proposed RSC-MOS. C-MOS employs a continuous gate structure. SG-MOS shortens the gate overlap above the JFET region to reduce the coupling between the gate and drift region. SC-MOS introduces a central P-type implanted region between the split-gates to shield the electric field at the split-gate edges. The central P^+^ region is formed simultaneously with the source-side P^+^ regions using the same implantation process and parameters, with the implantation windows defined at different lateral positions; therefore, no additional P^+^ implantation step is required. In RSC-MOS, the central P^+^ region is implanted on the preformed surface step, and the Schottky contact is subsequently formed along the stepped SiC surface. Based on SC-MOS, RSC-MOS raises the central implanted region toward the device surface and forms a stepped Schottky contact near this region. Here, “stepped” refers to the non-coplanar metal/SiC Schottky interface formed by the surface-height difference adjacent to the raised central implanted region, rather than a conventional planar Schottky interface. The step height represents the elevation of the central implanted region relative to that in SC-MOS. Raising this region reduces its occupation of the JFET conduction path, while the integrated Schottky contact provides a low-voltage-drop unipolar conduction path for third-quadrant current. The Schottky contact is electrically connected to the source electrode. The source metal forms Ohmic contacts with the heavily doped N^+^ and P^+^ regions, whereas the metal/N^−^ drift interface in the central stepped region forms the Schottky contact; such Ohmic/Schottky co-integration has been experimentally demonstrated using a single-metal process [16].

For a consistent comparison, all four devices employ a 10 μm thick drift region with a doping concentration of 8 × 10^15^ cm^−3^ to support a blocking voltage of 1200 V. They also use identical channel dimensions, gate oxide thicknesses, and implantation parameters. The Schottky contact was modeled with a metal work function of 4.33 eV, corresponding to a nominal Schottky barrier height of 1.09 eV for 4H-SiC. The same bias conditions and parameter extraction methods are applied to all devices. The device characteristics were simulated using Synopsys Sentaurus TCAD. The main physical models include Fermi statistics, incomplete ionization, doping- and temperature-dependent SRH recombination, Auger recombination, normal-field and high-field mobility models, and mobility anisotropy; the Okuto avalanche model was employed for breakdown simulation. Fixed charges and interface traps at the SiC/SiO_2_ interface were also considered. The channel length and gate oxide thickness were 0.5 μm and 0.05 μm, respectively, and implanted doping profiles were adopted for the doped regions. Adaptive mesh refinement was applied near the SiC/SiO_2_ interface and in regions with large doping gradients. The baseline TCAD framework follows our previous study of fabricated 1200 V 4H-SiC planar MOSFETs [32], in which the device structures were experimentally fabricated and characterized, and the corresponding TCAD simulations were established based on the fabricated device dimensions. The SiC/SiO_2_ interface-trap parameters were adjusted to reproduce the measured transfer characteristics of the baseline MOSFET. The resulting model parameters were then applied consistently to the comparative simulations of the four device structures.

**Figure 2:**
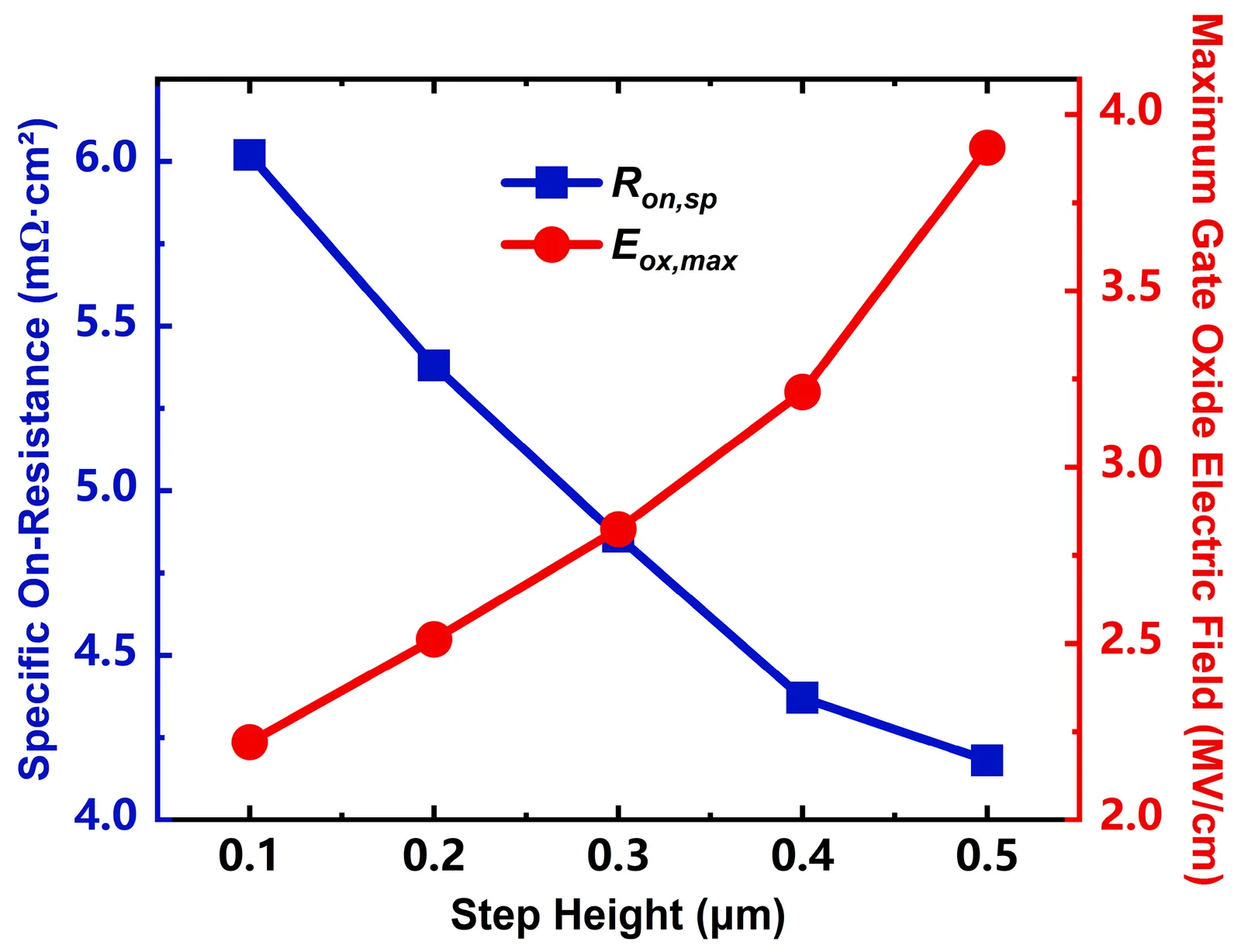
Effects of the central implanted region step height on R_on,sp_ and E_ox,max_ of RSC-MOS.

The elevation of the central implanted region directly affects the specific on-resistance and gate oxide electric field. Figure 2 shows the variations in specific on-resistance (R_on,sp_) and maximum gate oxide electric field (E_ox,max_) of RSC-MOS with the central implanted-region step height. As the step height increases, R_on,sp_ gradually decreases, whereas E_ox,max_ continuously increases. When the central P-type implanted region is located deeper in the JFET region, its depletion and constriction effects are stronger. This provides effective electric field shielding for the split-gate edges but narrows the available conduction region and restricts carrier transport through the JFET region. Raising the central implanted region reduces its occupation of the main current path and therefore lowers the R_on,sp_. However, the electric field shielding at the gate edges is weakened, resulting in a higher maximum gate oxide electric field.

Therefore, the step height cannot be selected solely to minimize the on-resistance. Based on the opposing trends of R_on,sp_ and E_ox,max_ in Figure 2, a step height of 0.33 μm was selected as a compromise between conduction resistance and the gate-oxide electric field. This value was directly simulated and was subsequently used for all RSC-MOS simulations presented in this work. The gradual variation in both parameters with step height also indicates that moderate process deviations do not cause abrupt performance degradation.

**Figure 3:**
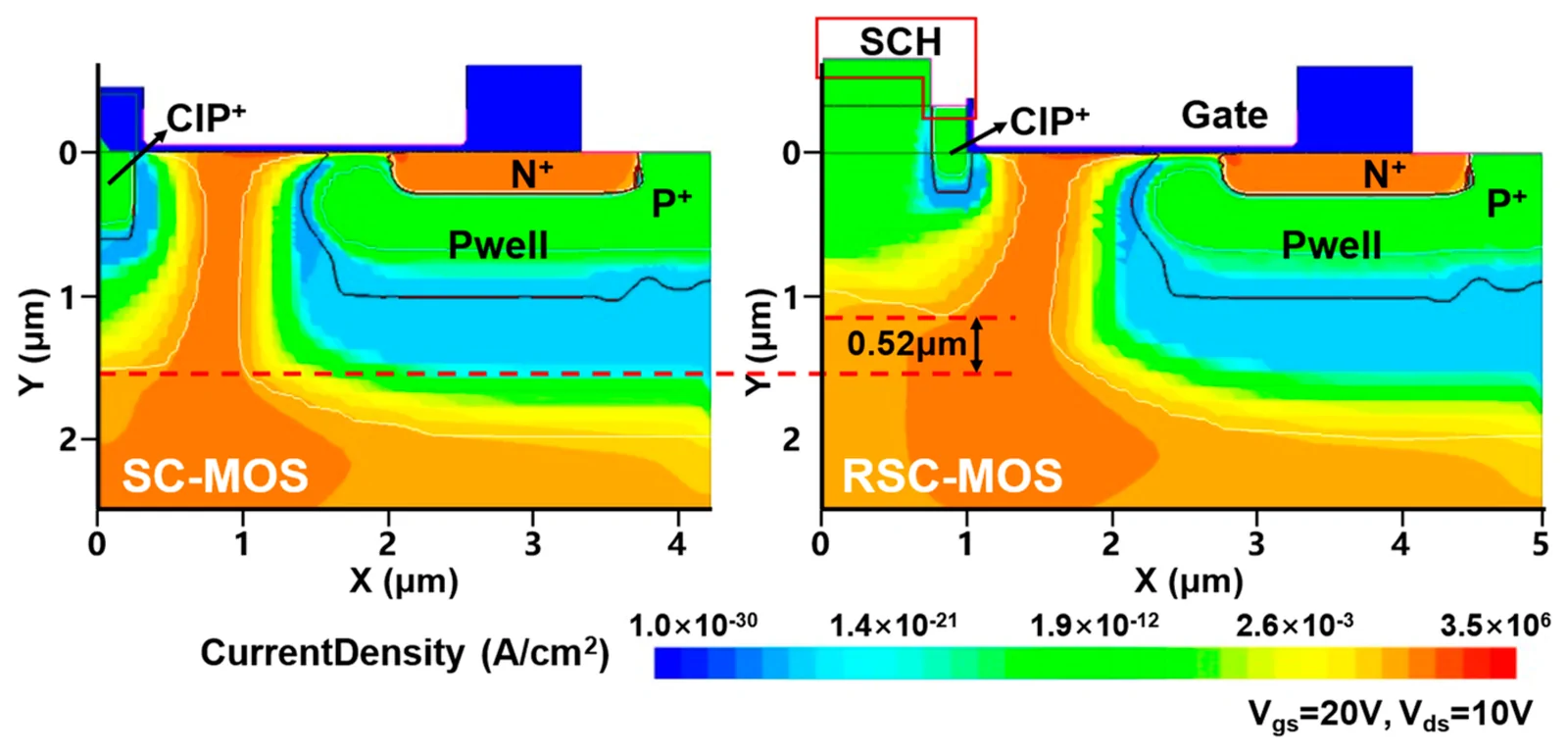
Current density distributions of SC-MOS and RSC-MOS under the same forward bias condition.

Figure 3 compares the current density distributions of SC-MOS and RSC-MOS under the same forward bias condition. In SC-MOS, the relatively deep central P-type implanted region occupies part of the JFET conduction region. Electrons flowing from the channel into the drift region must bypass this region, resulting in a narrowed current path and pronounced current crowding near the central implanted region. In RSC-MOS, raising the central implanted region increases the space available for electron transport beneath and along both sides of the central P-type region. As indicated by the red dashed lines in Figure 3, the depletion boundary in RSC-MOS is shifted upward by approximately 0.52 μm relative to that in SC-MOS. This produces a more continuous current path in the JFET region and reduces the restriction on current spreading. Therefore, the proposed structure retains the electric field shielding provided by the central implanted region while reducing the additional resistance caused by current path constriction in SC-MOS. Since the integrated Schottky diode is reverse-biased during forward conduction, the reduction in on-resistance mainly results from the change in the geometric position of the central implanted region.

**Figure 4:**
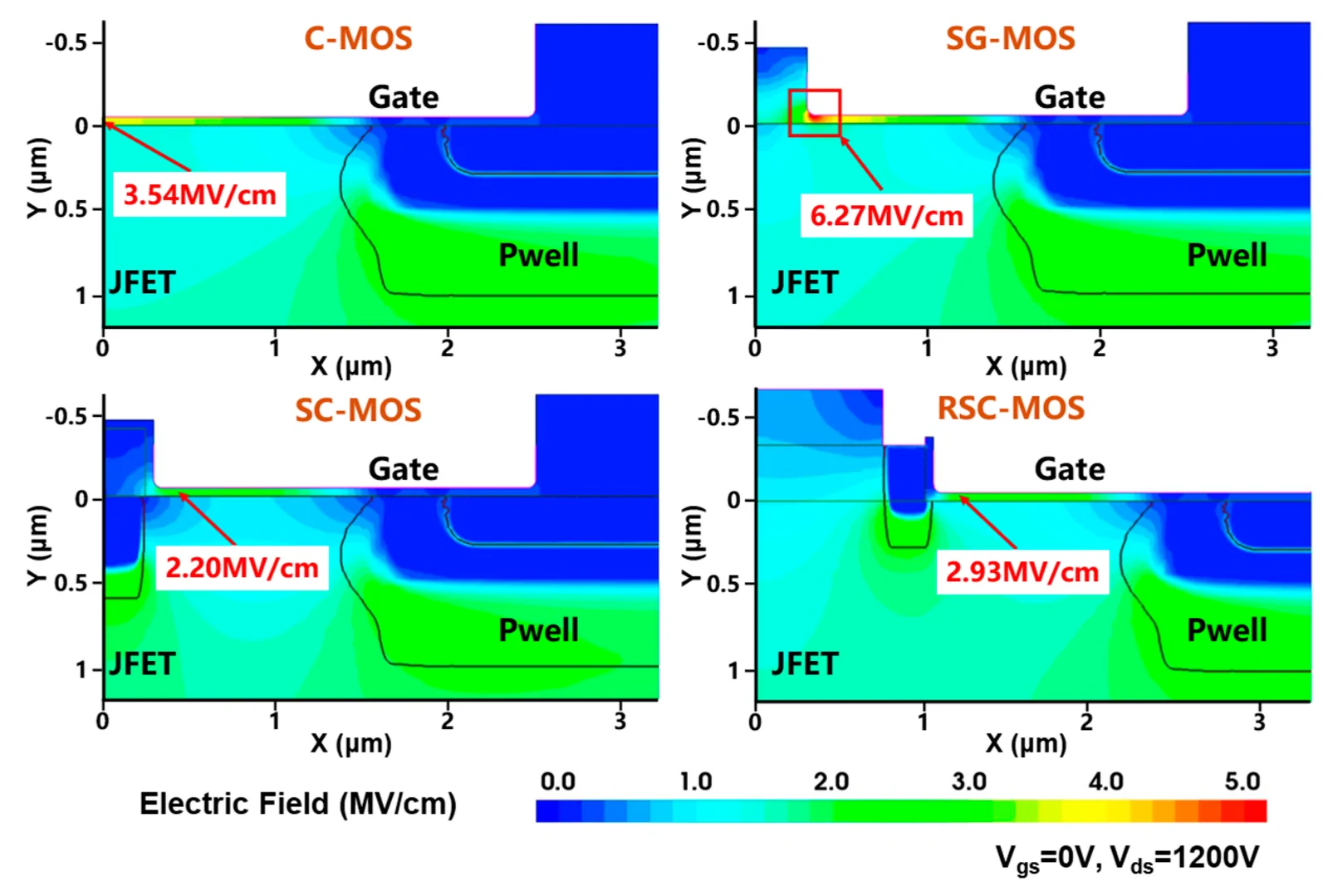
Gate oxide electric field distributions of the four devices under the same reverse-bias condition.

Figure 4 compares the gate oxide electric field distributions of the four devices under the same reverse bias condition. The maximum gate oxide electric field of C-MOS is 3.54 MV/cm. In SG-MOS, the gate edge directly faces the JFET region, resulting in more severe electric field crowding in the off state. Consequently, E_ox,max_ increases to 6.27 MV/cm. In SC-MOS, the central P-type implanted region forms a depletion region with the drift region in the off state, allowing part of the voltage to drop across the junction near the central implanted region. This significantly relieves the electric field at the split-gate edge and reduces E_ox,max_ to 2.20 MV/cm. However, the relatively deep implanted region also constricts the JFET current path and increases the on-resistance. The maximum gate oxide electric field of RSC-MOS is 2.93 MV/cm, which is 53.3% lower than that of SG-MOS and 17.2% lower than that of C-MOS. Although its shielding capability is weaker than that of SC-MOS, it still effectively suppresses electric field crowding at the split-gate edge and keeps E_ox,max_ below the commonly accepted limit of 3 MV/cm.

As shown in Figure 2 and Figure 3, raising the central implanted region moderately increases the gate oxide electric field but significantly reduces the restriction imposed by the central P-type region on the main current path. The proposed structure therefore achieves an improved tradeoff between the gate oxide electric field and on-resistance.

## 3. Simulation Results and Discussion

Based on the preceding analysis of the device structures and operating mechanisms, this section further compares the gate-charge, capacitance, and reverse characteristics of the four devices. In this work, the switching-related characteristics are evaluated mainly through the gate charge and reverse transfer capacitance.

**Figure 5:**
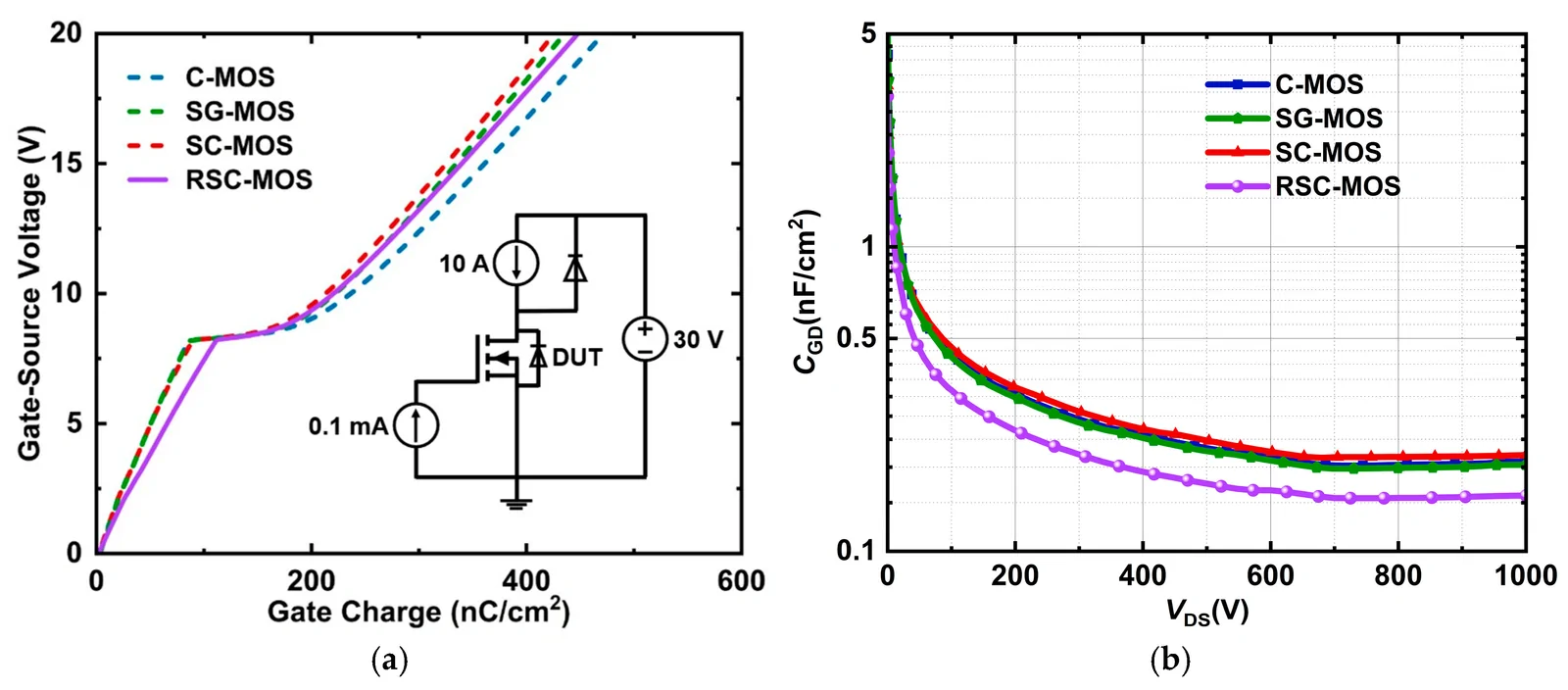
Gate-charge and capacitance characteristics of the four devices: (**a**) gate charge curves. (**b**) reverse transfer capacitance.

Figure 5a shows the gate-charge curves obtained at V_DS_ = 30 V and I_D_ = 10 A using a constant gate charging current of 0.1 mA. Q_gd_ was extracted from the charge interval of the Miller plateau, whose start and end points were determined from the intersections of linear fits to the pre-plateau, plateau, and post-plateau regions. The gate-to-drain charges (Q_gd_) of C-MOS, SG-MOS, SC-MOS, and RSC-MOS are 107.76, 94.41, 83.40, and 61.93 nC/cm^2^, respectively. Compared with C-MOS, SG-MOS, and SC-MOS, the Q_gd_ of RSC-MOS is reduced by 42.5%, 34.4%, and 25.7%, respectively. RSC-MOS therefore exhibits the shortest Miller plateau. The gate of C-MOS has a relatively large overlap with the JFET region, resulting in strong coupling between the gate and drift region. The split-gate structure shortens the gate length above the JFET region and thus reduces the gate-to-drain charge of SG-MOS. Compared with SC-MOS, the further reduction in Q_gd_ of RSC-MOS is mainly attributed to the source-connected Schottky anode introduced between the split-gates. The source-potential Schottky contact and its depletion region weaken the capacitive coupling between the gate and drift region, leading to a lower C_gd_ and consequently a smaller Miller charge. Figure 5b compares the reverse transfer capacitance (C_gd_) of the four devices. As the drain-to-source voltage increases, C_gd_ gradually decreases for all devices, while RSC-MOS maintains a relatively low value over the entire voltage range. This result agrees with the gate-charge characteristics and indicates that the proposed structure reduces the required gate-drive charge during the Miller interval.

To evaluate the overall tradeoff between conduction loss and gate-charge characteristics, the product of the specific on-resistance and gate-to-drain charge is used as the high-frequency figure of merit:

HFOM = R_on,sp_ × Q_gd_ (1)

where R_on,sp_ is the specific on-resistance and Q_gd_ is the gate-to-drain charge. A lower HFOM indicates reduced gate charging and discharging requirements while maintaining low conduction loss. The HFOM of RSC-MOS is 290.46 mΩ·nC, which is 36.7%, 28.0%, and 44.1% lower than those of C-MOS, SG-MOS, and SC-MOS, respectively. Although its R_on,sp_ is not the lowest among the four devices, the smaller Q_gd_ enables RSC-MOS to achieve the best tradeoff between conduction loss and gate-charge characteristics.

**Figure 6:**
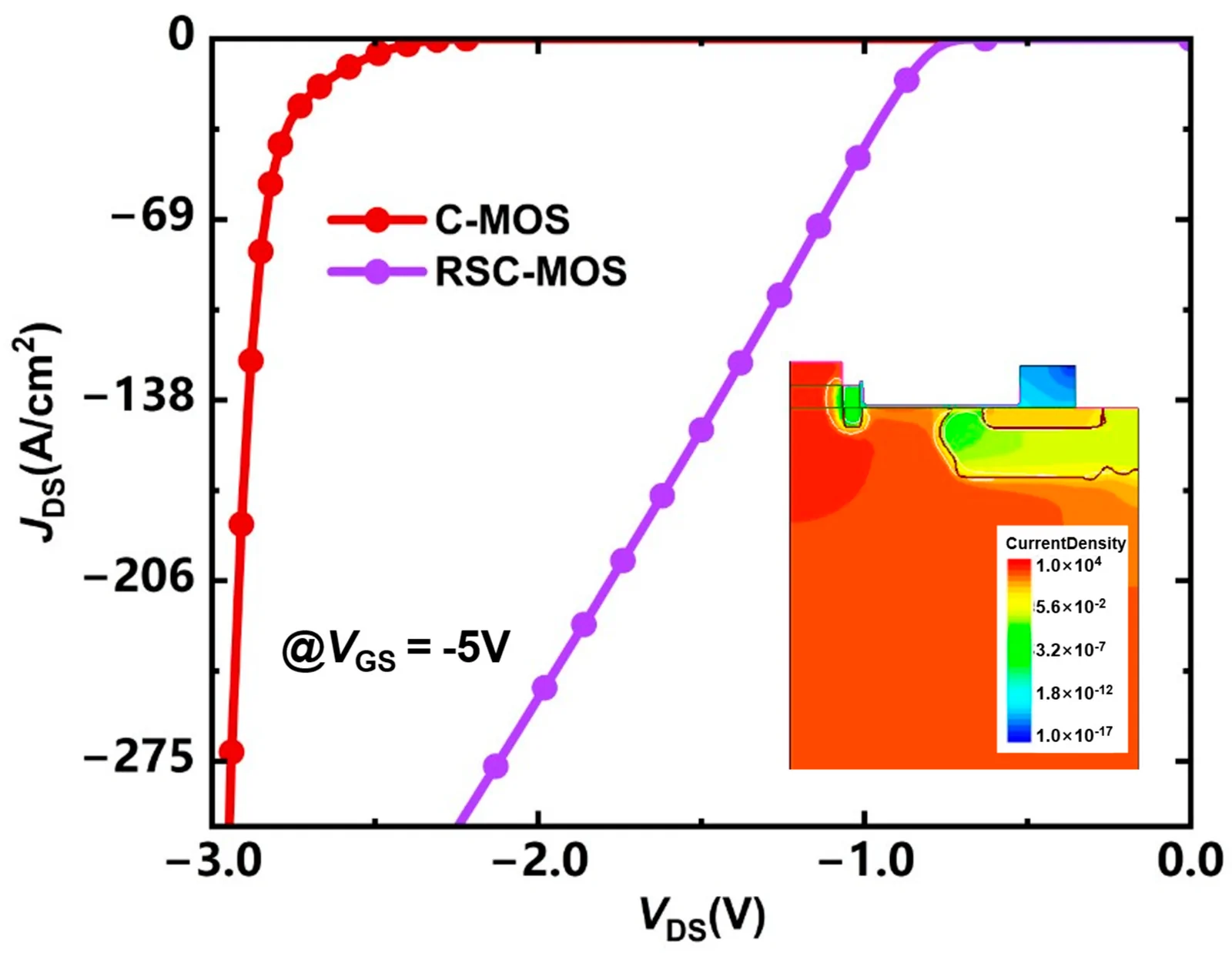
Third-quadrant characteristics at V_GS_ = −5 V and reverse conduction current density distribution of RSC-MOS.

Figure 6 shows the third-quadrant characteristics of C-MOS and RSC-MOS at VGS = −5 V. SG-MOS and SC-MOS also exhibit reverse conduction dominated by the parasitic PiN body diode, resulting in third-quadrant current-voltage characteristics nearly overlapping with those of C-MOS. C-MOS, SG-MOS, and SC-MOS mainly rely on their parasitic PiN body diodes for reverse conduction, whereas the integrated Schottky contact in RSC-MOS provides an additional unipolar conduction path. Under the same extraction criterion, the reverse turn-on voltage V_SD_ is 2.810 V for C-MOS and decreases to 1.042 V for RSC-MOS, corresponding to a reduction of 62.9%. The Schottky contact in RSC-MOS turns on at a lower reverse voltage and provides a unipolar conduction path for third-quadrant current. As shown by the current density distribution in Figure 6, the reverse current is mainly concentrated near the integrated Schottky contact, indicating that the Schottky barrier diode (SBD) conducts before the parasitic PiN body diode. The lower reverse turn-on voltage reduces conduction loss during the dead time and suppresses minority-carrier injection from the parasitic body diode.

**Figure 7:**
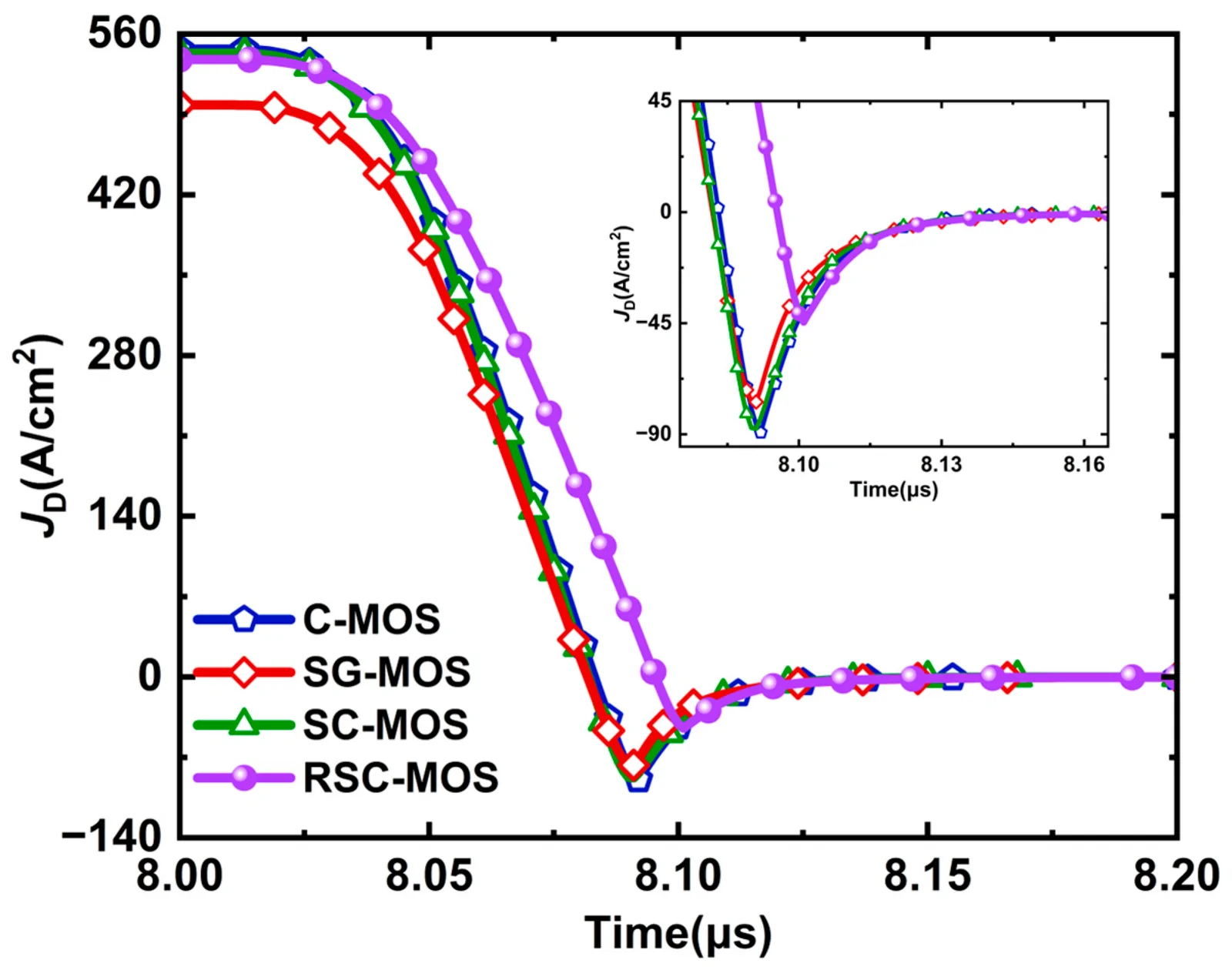
Reverse recovery current waveforms of the four devices.

Figure 7 compares the reverse-recovery current waveforms of the four devices using an inductive commutation circuit with a DC-link voltage of 100 V, a load inductance of 10 μH, a gate-drive voltage of 0–15 V, and a gate resistance of 100 Ω. C-MOS, SG-MOS, and SC-MOS exhibit relatively large reverse recovery current peaks. The split-gate structure of SG-MOS mainly modifies the gate related dynamic characteristics and does not significantly improve the reverse recovery behavior of the body diode. Although the reverse recovery charge of SC-MOS is slightly reduced, it remains relatively high. RSC-MOS exhibits a much lower reverse recovery current peak and a significantly smaller negative area enclosed by the current waveform. In this work, the reverse recovery charge per unit area is extracted by integrating the negative portion of the reverse recovery current density:

(2) $$Q_{\mathrm{rr}}=\int_{t_{0}}^{t_{1}}|J_{\mathrm{rr}}\left(t\right)|\;\mathrm{dt}$$

where t_0_ and t_1_ denote the beginning and end of the negative reverse recovery current interval, respectively, and J_rr_ is the reverse recovery current density. The Q_rr_ values of C-MOS, SG-MOS, SC-MOS, and RSC-MOS are 1459.08, 1495.70, 1338.14, and 676.00 nC/cm^2^, respectively. Compared with C-MOS, SG-MOS, and SC-MOS, the Q_rr_ of RSC-MOS is reduced by 53.7%, 54.8%, and 49.5%, respectively.

This improvement mainly results from the unipolar conduction of the integrated SBD. During reverse conduction, the Schottky path suppresses minority-carrier injection from the parasitic PiN body diode, thereby reducing the stored charge that must be removed during commutation. As a result, RSC-MOS reduces reverse recovery loss and helps alleviate the voltage and current stresses associated with reverse recovery current.

**Figure 8:**
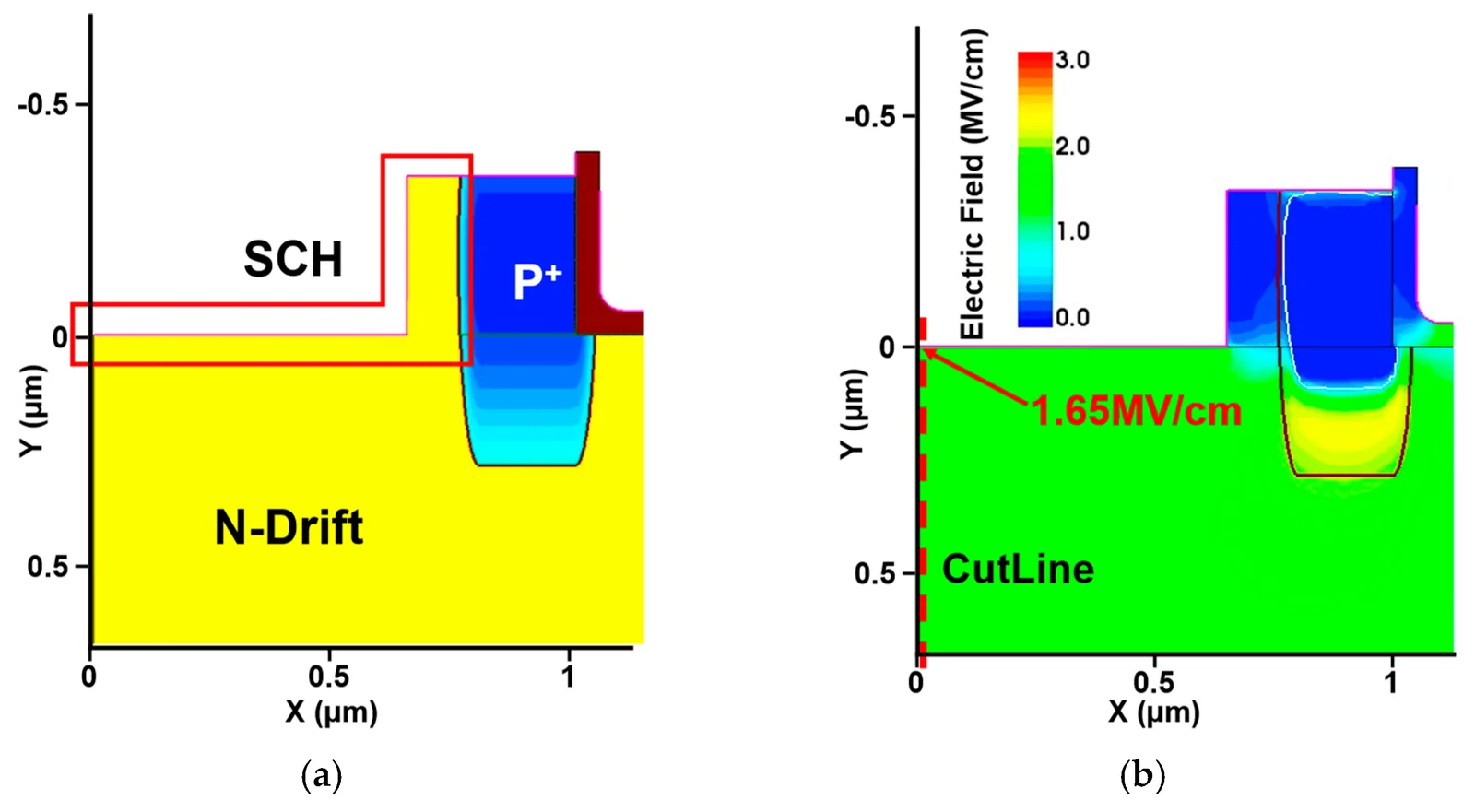

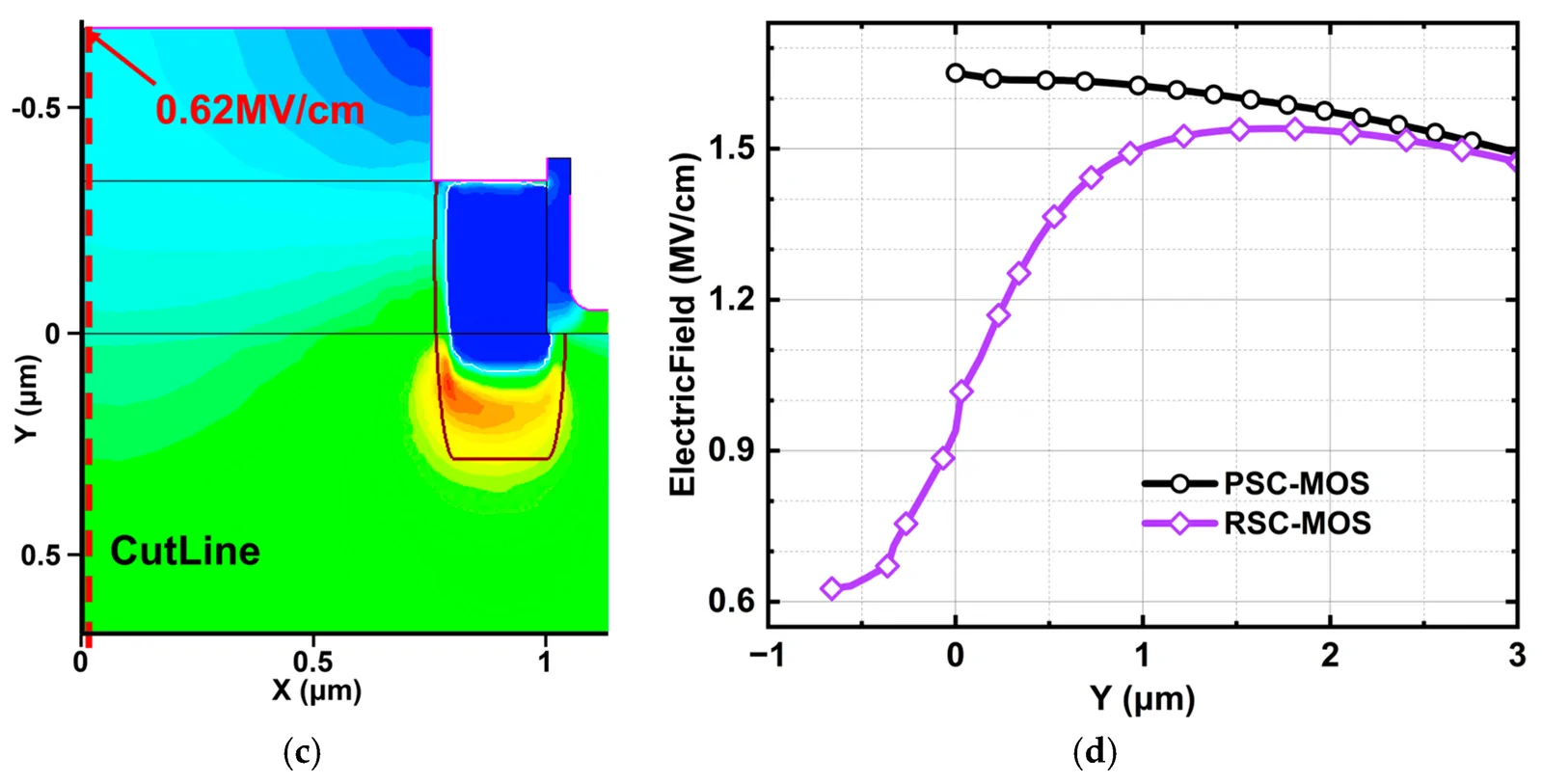
(**a**) Cross-sectional schematic of PSC-MOS. (**b**) Electric-field distribution of PSC-MOS. (**c**) Electric-field distribution of RSC-MOS. (**d**) Electric-field profiles along the vertical cutline.

To isolate the effect of the Schottky-contact geometry, PSC-MOS is introduced as a reference device with the same central P^+^ implantation profile as RSC-MOS. As shown in Figure 8a, the main horizontal Schottky contact of PSC-MOS is coplanar with the channel surface, while a short vertical Schottky-contact segment is retained adjacent to the central P^+^ region to facilitate a more consistent comparison with RSC-MOS. Figure 8b,c show the electric-field distributions of PSC-MOS and sRSC-MOS, respectively. Pronounced field crowding occurs near the beginning of the metal/SiC interface in PSC-MOS. As shown in Figure 8d, the electric field along the cutlines indicated in Figure 8b,c is 1.65 MV/cm for PSC-MOS and 0.62 MV/cm for RSC-MOS at the critical contact location. This corresponds to a reduction of 1.03 MV/cm, or approximately 62.4%. Although the P^+^ regions in PSC-MOS and RSC-MOS are identical, the stepped Schottky interface changes the two-dimensional electrostatic boundary condition near the contact. This spatially separates the Schottky interface from the high-field region around the P^+^/N^−^ drift junction corner, redistributing the local electric field from the Schottky contact onset toward the PN-junction corner. Although the electric fields of the two devices gradually approach each other away from the contact onset, RSC-MOS significantly suppresses field crowding at the critical contact location. This result indicates that the stepped geometry improves the off-state electric-field distribution near the Schottky contact.

Table 1 summarizes the main performance parameters of the four devices. RSC-MOS maintains a 1200 V blocking capability and a low gate oxide electric field while achieving the lowest high-frequency figure of merit, third-quadrant turn-on voltage, and reverse recovery charge among the four devices. Although its specific on-resistance is slightly higher than those of C-MOS and SG-MOS, it is substantially lower than that of SC-MOS. These results demonstrate that the proposed structure achieves a balanced tradeoff among blocking, conduction, gate-charge, and reverse characteristics.

## 4. Conclusions

A 1200 V split-gate 4H-SiC MOSFET featuring a raised central implanted region and a stepped Schottky contact is proposed and investigated through TCAD simulations. The proposed device reduces the specific on-resistance by 24.7% compared with SC-MOS and lowers the maximum gate oxide electric field by 53.3% compared with SG-MOS. Relative to C-MOS, its high-frequency figure of merit and reverse recovery charge are reduced by 36.7% and 53.7%, respectively. In addition, the stepped Schottky contact reduces the electric field at the critical contact location by 62.4%. These results demonstrate that RSC-MOS achieves a favorable tradeoff among conduction loss, gate oxide electric fields, gate-charge characteristics, and reverse recovery performance.

## Figures and Tables

**Table 1:** Main performance parameters of the four devices.

Characteristics	C-MOS	SG-MOS	SC-MOS	RSC-MOS
BV (V)	1487.68	1465.51	1493.09	1447.18
R_on,sp_ (mΩ·cm^2^)	4.26	4.27	6.23	4.69
E_ox,max_ (MV/cm)	3.54	6.27	2.20	2.93
Vth (V)	3.168	3.168	3.169	3.084
HFOM (mΩ·nC)	459.07	403.14	519.60	290.46
Q_gd_ (nC/cm^2^)	107.76	94.41	83.40	61.93
V_SD_ (V)	2.810	2.810	2.810	1.042
Q_rr_ (nC/cm^2^)	1459.08	1495.70	1338.14	676.00

Note: BV is extracted at I_D_ = 1 × 10^−5^ A. V_SD_ is extracted at J_SD_ = 50 A/cm^2^.

## Data Availability

The data presented in this study are available on request from the corresponding author.
